# Supplementary material for: Rapid turnover of life-cycle-related genes in the brown algae
Source: Genome Biol. 2019 Feb 14;20:35. doi: 10.1186/s13059-019-1630-6 (PMC6374913; doi:10.1186/s13059-019-1630-6)
Supplement: Supplementary file 3 — Protein alignments, corresponding Gblocks html file and CDS sequences for the lineage conserved gametophyte-biased genes. (ZIP 2109 kb) [file 13059_2019_1630_MOESM3_ESM.zip › Con_GA_alignments/Ec-20_001540.aa_ali.fasta-gb.htm]

Ec-20\_001540.aa.fasta.trans.aa\_ali.fasta


## Gblocks 0.91b Results

Processed file: **Ec-20\_001540.aa.fasta.trans.aa\_ali.fasta**  
Number of sequences: **2**  
Alignment assumed to be: **Protein**  
New number of positions: **457** (selected positions are underlined in blue)

```
                         10        20        30        40        50        60
                 =========+=========+=========+=========+=========+=========+
Ec-20_001540     MNKYEVLGVVGEGAYGVVLRCRNKDSGCVVAVKKFKESEDDEIVKKTTLREVKVLRMLRH
MSTRG.33002.1    --------------------------------------------------------MLRH
                                                                         ####


                         70        80        90       100       110       120
                 =========+=========+=========+=========+=========+=========+
Ec-20_001540     PNIVCLKEAFRRKGKLYLVFEYVEKNLLEVLEDNPDGLPFEAVRGYIHQLCRAIDWCHHN
MSTRG.33002.1    PNIVCLKEAFRRKGKLYLVFEYVEKNLLEVLEDNPDGLPFEAVRGYIHQLCRAIDWCHHN
                 ############################################################


                        130       140       150       160       170       180
                 =========+=========+=========+=========+=========+=========+
Ec-20_001540     GVVHRDIKPENLLVNARSNELKLCDFGFARIVPPSSAHRQELTDYVATRWYRAPELLLGS
MSTRG.33002.1    GVVHRDIKPENLLVNARSNELKLCDFGFARIVPSSSGHRQELTDYVATRWYRAPELLLGS
                 ############################################################


                        190       200       210       220       230       240
                 =========+=========+=========+=========+=========+=========+
Ec-20_001540     TRYDPSVDLWAIGCIMGELVDGQPLFPGDSEIDQLYIVQRVLGPLTEAHMQLFLKNPRFV
MSTRG.33002.1    TRYDPSVDLWAIGCIMGELVDGQPLFPGDSEIDQLYIVQRMLGPLTEAHLQLFLKNPRFV
                 ############################################################


                        250       260       270       280       290       300
                 =========+=========+=========+=========+=========+=========+
Ec-20_001540     GLKFPDMSRPETLQKRFVGKLGKRAMAFVRSSLAMDPAARVSSREAVCHPLFEGLFEDYA
MSTRG.33002.1    GLKFPDMSRPETLQKRFVGKLGKRAMAFMRSALTMDPAVRVSSRDAVCHPLFEGLFEDYA
                 ############################################################


                        310       320       330       340       350       360
                 =========+=========+=========+=========+=========+=========+
Ec-20_001540     SRHPHLQQRGDDLGGGVGGSANGSGGGLQNGSSGNGGGVGGGAAVVGAGGDGHNRDDTRG
MSTRG.33002.1    SRHPHLHQCGDNVGG----TATAGTGALQGG---TGSGNGGGQAGISGGGG--GRDGGRG
                 ###############     ###########    ###############    ######


                        370       380       390       400       410       420
                 =========+=========+=========+=========+=========+=========+
Ec-20_001540     TGGGGRGTSNHQAPAQQHGNGSSANGGGSSGRPTDLQGGEDAHQTPGPKPPSGGVPRLGR
MSTRG.33002.1    TGAGGSTMMDHQTAGQQHGPGAGVDGGSLSGR-HPSQGGTDAQQSSGPKPPSGGVPRLGR
                 ################################    ########################


                        430       440       450       460       470       480
                 =========+=========+=========+=========+=========+=========+
Ec-20_001540     FTRQGSQQHQQQQQQQQQQQQQGAHYSQRSSGGSDLKSSFQDGERGSGRSGSVSNSWGHS
MSTRG.33002.1    FNRQGSQ------QQQQQPLQQNNHHNQRSSGG-DPKGFQQDGERWGGRGSAANGNSGGS
                 #######      #################### ##########################


                        490       500       510       520       530       540
                 =========+=========+=========+=========+=========+=========+
Ec-20_001540     KGGGWEGRAGTGGGGGGGGGGSNGGNRRASG--HAGQGNGLTRHSRDQGRHSSSREDGGR
MSTRG.33002.1    NAGGKG--SGWDGRGGAVAGGSNG-HRRSSGSSNGGQGNGASRYGRENGRQATGREDGIR
                 ####     ###############           #########################


                        550       560       570       580       590       600
                 =========+=========+=========+=========+=========+=========+
Ec-20_001540     AGGGGGGNGDGGTGNGGGGGGNGGRRAGGRGGRDSSGGKGRYGGGGGAGGRGARAPATSA
MSTRG.33002.1    ASG--CGNGDGGAGNG--------------------------------------------
                 ###   ##########                                            


                        610       620       630       640       650       660
                 =========+=========+=========+=========+=========+=========+
Ec-20_001540     AGNSVDNNIGRSGSPIHSGRHHSNGNHRDSGLIYGGEGGQYYNNNNNNNNNNNGSNGHYH
MSTRG.33002.1    ------------------------------------------------------------
                                                                             


                        670       680       690       700       710       720
                 =========+=========+=========+=========+=========+=========+
Ec-20_001540     HQQQQHQPSQQAHRQNHHHHHQQQQQQQQPFLSSSCRDEQQWQNGTPQLPGFARNGDGGG
MSTRG.33002.1    ------------------------------------------------------------
                                                                             


                        730       740       750       760       770       780
                 =========+=========+=========+=========+=========+=========+
Ec-20_001540     GGGDGGDDRDRKGHGYGTSRDGRDRESKESTRDRGRYRDGGGGGGGGSRDWSRGDFGGRD
MSTRG.33002.1    ------------------------------------------------------------
                                                                             


                        790       800       810       820       830       840
                 =========+=========+=========+=========+=========+=========+
Ec-20_001540     SSREASNRDGGSGGGGSGRRGPAPLRGRHDDLGAYAEPKASPRGGGRFSPGDGGMKGVSP
MSTRG.33002.1    ------------------------------------------------------------
                                                                             


                        850       860       870       880       890       900
                 =========+=========+=========+=========+=========+=========+
Ec-20_001540     RLNGGGAATGRSMAAAGGRGAEGKPPNTRRWKGDGGGQGAAAEEKRQLEREIQAERERQR
MSTRG.33002.1    ------------------------------------------------------------
                                                                             


                        910       920       930       940       950       960
                 =========+=========+=========+=========+=========+=========+
Ec-20_001540     ENEIRAFRDFSLNLSSTPLPSALGGQGGDDHDDAGFPRGPPKRAEYKTGVGVNLDALRDP
MSTRG.33002.1    ------------------------------------------------------------
                                                                             


                        970       980       990      1000      1010      1020
                 =========+=========+=========+=========+=========+=========+
Ec-20_001540     NLAPRSRGRDNDPLTGGRDNLAPTGGIGVGGSGGTGNALGPGGGGGGGVGGGVGRRLFSV
MSTRG.33002.1    ------------------------------------------------------------
                                                                             


                       1030      1040      1050      1060      1070      1080
                 =========+=========+=========+=========+=========+=========+
Ec-20_001540     GGGGGAGCGAVGGVGNGGDVSGGGSSPPTASARYPPLEHIQNPPQPDPKAVADMDKFPGP
MSTRG.33002.1    ------------------------------------------------------------
                                                                             


                       1090      1100      1110      1120      1130      1140
                 =========+=========+=========+=========+=========+=========+
Ec-20_001540     HSSGGGSARGSGGSIIGKTSPLNGCYPMSPLHVKRRSSSNPNSNSSSGGLGGAGLGEEKM
MSTRG.33002.1    ------------------------------------------------------------
                                                                             


                       1150      1160      1170
                 =========+=========+=========+==
Ec-20_001540     EAKGGGGMGMGMGMGSDNLDAGYLPQLRRSEG
MSTRG.33002.1    --------------------------------
```

```
Parameters used
Minimum Number Of Sequences For A Conserved Position: 2
Minimum Number Of Sequences For A Flanking Position: 2
Maximum Number Of Contiguous Nonconserved Positions: 8
Minimum Length Of A Block: 10
Allowed Gap Positions: None
Use Similarity Matrices: Yes
```

```
Flank positions of the 10 selected block(s)
Flanks: [57  315]  [321  331]  [336  350]  [355  392]  [397  427]  [434  453]  [455  484]  [490  504]  [516  543]  [547  556]  

New number of positions in Ec-20_001540.aa.fasta.trans.aa_ali.fasta-gb:  457  (38% of the original 1172 positions)
```
